# Assessing the Levels and Types of Bacterial Contamination in Cosmetic Brushes: Implications for Beauty and Hygiene in Jeddah City

**DOI:** 10.1155/ijm/2128581

**Published:** 2025-05-06

**Authors:** Roba M. S. Attar, Mohammed A. Imam

**Affiliations:** ^1^Department of Biological Sciences, University of Jeddah, College of Science, Jeddah, Saudi Arabia; ^2^Department of Medical Microbiology and Parasitology, Umm Al-Qura University, Al-Qunfudah College of Medicine, Makkah, Saudi Arabia

**Keywords:** bacterial contamination, cosmetics, hygiene, skin problems

## Abstract

Cosmetic tools, such as brushes and sponges, are integral to beauty routines but are often neglected in terms of hygiene, posing risks of bacterial contamination and related skin issues. This study investigates bacterial contamination in 57 cosmetic brushes collected from users in Jeddah, Saudi Arabia. Bacterial isolates were characterized morphologically and identified through 16S rRNA gene sequencing. The results revealed a diverse microbial profile, with Gram-positive bacteria predominating (81%), including *Staphylococcus* and *Micrococcus* species, alongside Gram-negative bacteria such as *Pseudomonas* spp. A survey of 370 participants highlighted inconsistent cleaning habits, with 44.3% rarely cleaning their brushes and 27.8% reporting skin problems potentially linked to contaminated tools. A statistical analysis revealed significant correlations between awareness of hygiene practices and concerns about bacterial infections (*p* < 0.05), yet no direct association was found between cleaning frequency and skin issues (*p* = 0.698). This study emphasizes the need for public education on the proper maintenance of cosmetic tools to minimize health risks and promote safer beauty practices.

## 1. Introduction

Cosmetics, which are widely used for beauty enhancement, sun protection, and removing impurities, have become an integral part of everyday life [1]. These essential tools, which are used daily by millions, come into direct contact with our skin and makeup products, potentially harboring and transferring bacteria. Cosmetic products contain essential minerals and chemical compounds in water, which can create favorable environments for microbial growth [2]. Many individuals who use and share tools in beauty shops are unaware that makeup tools can harbor various microorganisms, posing a risk of exposure to potentially infectious agents [3]. If not handled properly, cosmetics can transmit skin or eye infections to and between clients. Therefore, cleanliness and hygiene are paramount, yet one often overlooked aspect of beauty routines is the maintenance of cosmetic brushes. The relationship between bacterial contamination and cosmetic brushes is a growing concern, as improper care and hygiene practices can lead to the accumulation of harmful microbes.

Microbiological studies consistently highlight bacterial contamination in cosmetic tools. *Streptococcus*, *Staphylococcus*, and *Pseudomonas* genera are of particular concern because they are associated with numerous common infections and can cause respiratory issues and antimicrobial-resistant infections due to their pathogenic nature [4–7]. Surveys on personal toiletries show that *Bacillus*, *Staphylococcus* sp., *Pseudomonas* sp., *Enterobacter*, *Aspergillus*, *Penicillium*, and *Candida* are prevalent in cosmetics [3]. *Staphylococcus* species, particularly *Staphylococcus aureus*, were detected at high rates, with 37% prevalence [8] and up to 100% in some tools [9]. *Pseudomonas aeruginosa* was also identified at high frequencies, ranging from 69.6% to 81.8% [9]. Other bacterial species such as *Streptococcus* sp. (12%) and *Enterococcus* sp. (5%) were reported at lower rates. Additionally, fungal contamination was a notable concern, with *Candida albicans* found in 13% of samples [8] and other fungi detected in 20.5%–51.5% of samples [9–11].

In general, most cosmetic brushes and other beauty tools, even after being thoroughly sanitized, still pose a risk of bacterial transmission and contamination whenever they come into contact with breaks in the skin [3, 12–14]. Numerous studies have highlighted the issue of bacterial contamination on cosmetic brushes, emphasizing the importance of hygiene in beauty practices. Research indicates that cosmetic tools can serve as reservoirs for various microorganisms, including bacteria and fungi, some of which pose potential health risks. Pathogens such as *Staphylococcus aureus*, *Klebsiella pneumoniae*, and *Pseudomonas aeruginosa* are of particular concern due to their association with skin infections and opportunistic diseases. Additionally, fungal contaminants like *Candida albicans* and *Aspergillus* spp. may contribute to allergic reactions and dermatological conditions. The presence of these microorganisms underscores the importance of proper hygiene practices, as contamination can occur through prolonged use, improper storage, and infrequent cleaning. Understanding the microbial load on beauty tools is crucial for mitigating health risks and promoting safer cosmetic practices [1, 3, 15, 16].

Microbial contamination is a significant concern across various everyday materials and surfaces, including medical tools, personal hygiene products, and household objects. Research has shown that bacterial pathogens can persist on diverse materials such as polymeric surfaces, textiles, and even electronic devices, increasing the risk of cross-contamination and infections. For instance, studies on protective facemasks made of nonwoven fabric have demonstrated that their surface properties influence microbial adhesion, with factors like hydrophobicity and electron donor/acceptor characteristics affecting bacterial attachment [17, 18]. Similar concerns extend to cosmetic brushes, which provide a suitable environment for microbial growth due to repeated use, moisture retention, and direct skin contact. Understanding contamination dynamics in beauty tools is crucial for mitigating potential health risks and improving hygiene standards in the cosmetic industry.

Exposure to skin oils, makeup residue, moisture, improper storage conditions, and growth factors present in these environments often contributes to the accumulation of bacteria on brushes, creating a conducive environment for microbial growth [19]. Although the level of risk associated with these applicators depends on their structure, composition, and configuration, those with the highest risk of contamination transfer are those capable of trapping and retaining moisture, dirt, skin cells, and microorganisms [20]. Makeup applicators can be easily contaminated through sharing or repetitive use, especially since the microflora of the skin are unique and can be transferred to others, potentially threatening users' health [20]. While commercially available cleaning products can reduce microbial contamination in cosmetic applicators to some extent, most makeup brushes and other objects can still pose a risk, especially when they come into contact with breaks in the skin [3, 13].

This study provides a comprehensive assessment of bacterial contamination in cosmetic brushes used by the general public and professional makeup artists in Jeddah City, identifying the specific bacterial species present and evaluating potential health risks. Unlike previous studies, our research uniquely correlates contamination levels with user habits, brush material, and storage conditions, offering new insights into hygiene practices in the beauty industry.

## 2. Methodology

### 2.1. Sample Collection (Cosmetic Tools)

Samples (*n* = 57) of cosmetic brushes (foundation brushes, eye makeup brushes, and powder brushes) and beauty blenders were collected in sterile bags with the approval and consent of makeup owners. The sample size was chosen based on the practical availability of cosmetic tools across various makeup owners in different regions of Jeddah City. To prevent secondary contamination, all samples were immediately sealed and transported in a temperature-controlled insulated container to maintain stability. Upon arrival at the research laboratory at the College of Science, University of Jeddah, Jeddah, Saudi Arabia, the samples were stored at 4°C until processing. We believe that the sample size of 57 is representative, as it captures a diverse range of cosmetic tools commonly used by the population, including both high- and low-use items, which are critical for understanding bacterial contamination. The samples were streaked on nutrient agars (NAs) and blood agars (BAs) before overnight incubation at 37°C at the research laboratory at the College of Science, University of Jeddah, Jeddah, Saudi Arabia.

### 2.2. Isolation and Purification of Bacteria

Colonies grown on NAs or BAs were separated by means of subculturing on fresh agars for purification. Single colonies were first characterized according to their morphological appearance, including their shape, margin elevation, size, surface, color, and optical properties.

The cosmetic brushes and beauty blenders were rubbed directly onto the surface of NA and BA plates to transfer the bacterial samples. All work was carried out in a biosafety cabinet to maintain sterile conditions. The plates were then incubated at 37°C overnight. After incubation, individual colonies grown on NAs or BAs were isolated and purified by means of subculturing on fresh NAs and BAs. The isolated single colonies were subsequently characterized based on their morphological appearance, including shape, margin elevation, size, surface, color, and optical properties.

### 2.3. Identification of Bacteria (16S)

Bacteria were identified through the amplification and sequencing of 16S ribosomal RNA genes using polymerase chain reaction (PCR) with a Techne Prime Thermal Cycler at the research laboratory of Al-Qunfudah College of Medicine, Al-Qunfudah, Saudi Arabia.

#### 2.3.1. DNA Extraction and Colony PCR

For bacterial DNA extraction, a well-isolated colony was transferred into 50 *μ*L of sterile water and boiled for 10 min. Then, the mixture was centrifuged at 16,000 × *g* for 5 min, and 5 *μ*L of the supernatant was used as a template for PCR. Each PCR was composed of 4 *μ*L of FIREPol Master Mix (Solis BioDyne, Estonia), 0.6 *μ*L of forward primer F27 (AGAGTTTGATCCTGGCTCAG), 0.6 *μ*L of reverse primer P2 (ACGGCTACCTTGTTACGACTT), 5 *μ*L of DNA template, and 9 *μ*L of ultrapure water. An Eppendorf vapo protect thermal cycler (Germany) was used for the amplification with the following steps: initial denaturation (95°C for 3 min), followed by 30 cycles of denaturation (95°C for 15 s), annealing (55°C for 30 s), and extension (72°C for 40 s) before the final extension step for 5 min at 72°C.

#### 2.3.2. Gel Electrophoresis

We prepared 1% agarose gel by adding 1 g of agarose powder to 100 mL of 1X TAE buffer in a flask. The mixture was heated in a microwave until the agarose dissolved completely and was then allowed to cool to 60°C. We added 1 *μ*L of GelRed to the gel mixture and poured the mixture into a gel casting tray with a comb inserted. The gel was allowed to solidify for 30 min at room temperature. We mixed 2 *μ*L of loading dye with 5 *μ*L of the PCR product and loaded the mixture into the wells of the gel using a micropipette. We also loaded 5 *μ*L of DNA ladder into one of the wells as a size reference. The gel was run in an electrophoresis apparatus for 45 min at 100 V. After the run was complete, the gel was removed from the apparatus and placed on a UV transilluminator. The DNA bands were visualized with UV light and photographed using a gel documentation system.

#### 2.3.3. 16S Ribosomal RNA Sequencing and Bacterial Identification

The purity and concentration of amplicons were determined using NanoDrop. The samples were sent to Macrogen Inc. (Republic of Korea) for 16S ribosomal RNA gene sequencing. The resulting sequences were submitted to the NCBI Nucleotide Blast platform to find homologous genes in bacterial genomes in the database.

### 2.4. Survey

A questionnaire was designed to gather data on makeup brush usage, cleaning habits, and related skin issues among various demographic groups. The first section collected demographic information, including age, gender, and education level, to enable demographic-specific analysis. The second section focused on makeup brush usage, querying the frequency and types of brushes used, cleaning habits, and any related skin issues or infections. The third section assessed respondents' knowledge and concerns about bacterial contamination in makeup brushes and their awareness of the importance of regular cleaning. The questionnaire included both closed-ended and multiple-choice questions to facilitate quantitative analysis. It was distributed online via Google Forms to reach a diverse and broad audience. Anonymity and confidentiality were ensured to encourage honest and unbiased responses. Data were analyzed using statistical software to identify trends, patterns, and correlations among the variables of interest.

### 2.5. Ethical Considerations

IRB approval was obtained from the biomedical research ethics committee at Umm Al-Qura University (HAPO-02-K-012-2023-12-1918).

### 2.6. Statistical Analysis

Descriptive statistics were used to summarize and describe the characteristics of the study participants and other categorical variables. Frequencies and percentages were calculated for categorical variables. Additionally, the Pearson chi-square test was conducted to find associations between the categorical variables. Simple and cluster bar charts were used for data visualization. The significance level for all statistical tests was set at *p* < 0.05, indicating a 95% confidence interval. All statistical calculations were performed using IBM SPSS Version 27.0.1.

## 3. Results

### 3.1. Isolation of Bacteria

**Figure 1:**
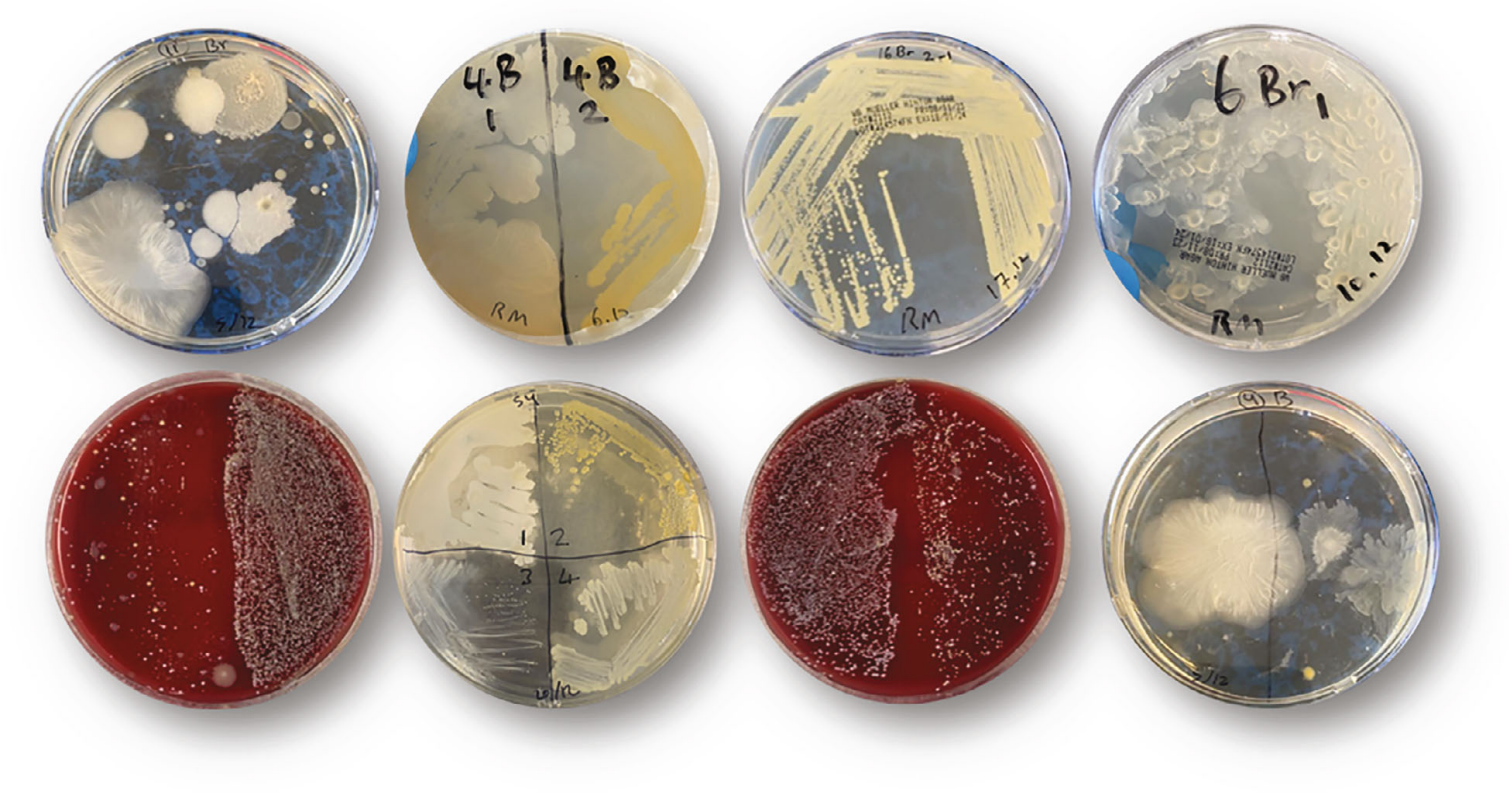
The growth of various bacterial colonies on nutrient and blood agars, demonstrating distinct morphological characteristics, including variations in shape, color, size, and structure. The diversity in colony appearance highlights the presence of multiple bacterial species, indicating a complex microbial community on cosmetic brushes.

Collected samples were streaked on NA or BA and incubated overnight at 37°C. The next day, the plates were observed for bacterial growth. The plates showed the growth of bacteria with different shapes, colors, sizes, and structures (Figure 1). The different agar media used support the growth of both general and selective bacteria, aiding in the identification of potential pathogens. The observed colony morphologies correspond to bacterial species isolated in the study, emphasizing the contamination risks associated with inadequate cosmetic brush hygiene.

**Figure 2:**
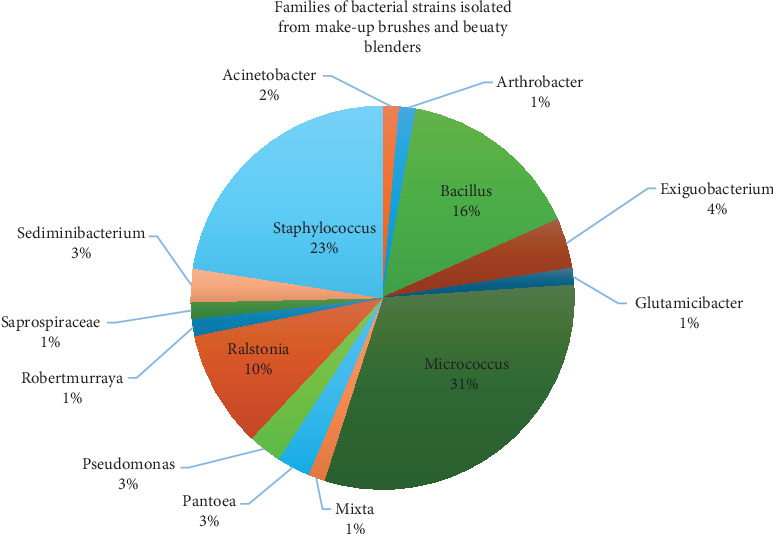
The bacterial families found on used makeup brushes and beauty blenders, with *Micrococcus*, *Staphylococcus*, and *Bacillus* being the most common. These bacteria, including *Staphylococcus aureus* and *Micrococcus luteus*, are linked to skin infections. Other identified families, such as *Ralstonia* and *Pseudomonas*, indicate potential health risks, highlighting the importance of proper hygiene in cosmetic tool maintenance.

A total of 71 strains were collected and identified according to their morphological appearance and according to 16S rRNA gene amplification and sequencing. Most isolates were Gram-positive (81%), and only 19% of the isolates were Gram-negative. The bacterial analysis of used makeup brushes and beauty blenders revealed a diverse microbial composition, with *Micrococcus* (31%) and *Staphylococcus* (23%) being the dominant families. These bacteria, commonly found on the skin, may contribute to dermatological issues when hygiene practices are inadequate. *Bacillus* (16%) and *Ralstonia* (10%) were also frequently detected, indicating potential environmental contamination. The presence of *Pseudomonas* (3%) and *Acinetobacter* (2%) is of concern due to their association with opportunistic infections (Figure 2).

The bacterial isolates were identified from cosmetic brushes, representing diverse genera (Table 1). *Staphylococcus* was the most prevalent, with 16 isolates, including *Staphylococcus aureus*, *Staphylococcus epidermidis*, and *Staphylococcus haemolyticus*, known for their role in skin infections. *Micrococcus* (22 isolates) and *Bacillus* (11 isolates) were also commonly detected, with species like *Micrococcus luteus* and *Bacillus cereus*, which can contribute to opportunistic infections. Other notable genera included *Pseudomonas*, *Ralstonia*, *Acinetobacter*, and *Pantoea*, some of which are associated with antimicrobial resistance. These findings highlight the potential health risks posed by contaminated cosmetic tools and emphasize the need for proper hygiene practices. The strains of these bacteria are listed in Table 1.

### 3.2. Questionnaire

**Figure 3:**
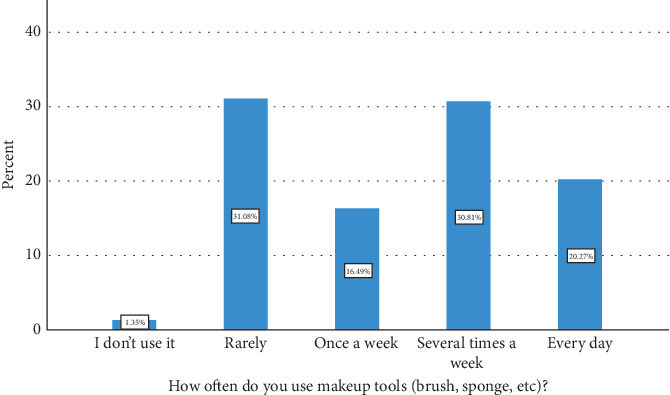
Frequency of makeup brush usage.

**Figure 4:**
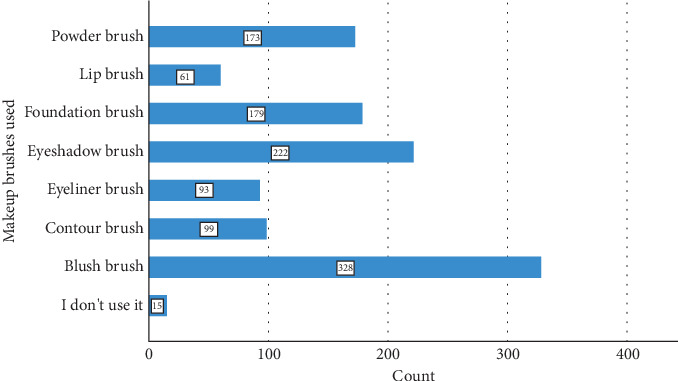
Makeup brushes used.

The sociodemographic profile of the study participants is detailed in Table 2. The age distribution revealed a significant presence of participants aged 35–54 years, with 28.9% (107 participants) falling into the 35–44-year-old category and 21.1% (78 participants) falling into the 45–54-year-old category. Younger age groups, including those under 18 years (3.0% with 11 participants) and those aged 18–24 years (11.6% with 43 participants), also contributed notably to the study cohort. Participants aged 55–64 years constituted 16.8% (62 participants), while those over 65 years represented 4.3% (16 participants). In terms of education, the majority of the participants held bachelor's degrees, comprising 52.7% (195 participants) of the sample. Those with a high school education or its equivalent accounted for 23.2% (86 participants), while individuals with master's degrees made up 11.1% (41 participants). Smaller segments included those with doctorates (5.1% with 19 participants), educational qualifications below secondary school (5.1% with 19 participants), and specialized qualifications such as fellowships or medical board certifications (1.1% with four participants). The “other” category represented 1.6% (six participants) of the total.  Table 3 and Figures 3 and 4 summarize the type and frequency of use of makeup brushes. A notable portion of respondents reported rarely using makeup tools, accounting for 31.1% (115 participants). In addition, 20.3% (75 participants) used makeup tools every day, 30.8% (114 participants) used them several times per week, 16.5% (61 participants) used them once per week, and a small percentage, 1.4% (five participants), reported not using makeup tools at all. Regarding the specific types of makeup brushes used, the most commonly utilized were blush brushes, with 88.6% (328 participants) reporting their use. Following closely were powder brushes, which were used by 46.8% (173 participants), and eyeshadow brushes, which were used by 60.0% (222 participants). Foundation brushes were utilized by 48.4% (179 participants), while contour brushes were used by 26.8% (99 participants). Eyeliner brushes and lip brushes were used by 25.1% (93 participants) and 16.5% (61 participants), respectively. A small proportion, 4.1% (15 participants), reported not using any makeup brushes.

**Figure 5:**
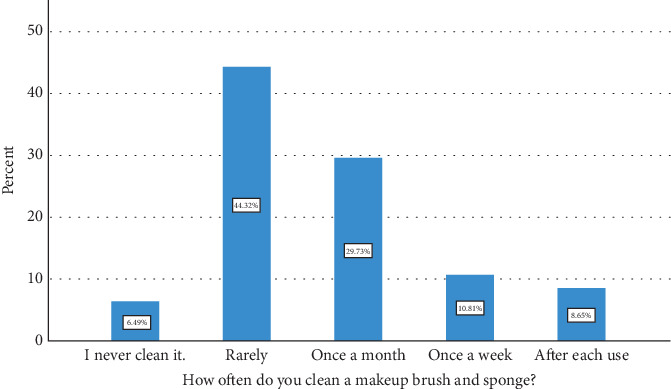
Frequency of makeup brush cleaning.

Table 4 and Figure 5 reveal the varied cleaning habits among participants regarding makeup brushes and sponges. A minority, comprising 8.6% (32 participants), reported cleaning these tools after each use, while 6.5% (24 participants) admitted to never cleaning them. A significant portion, amounting to 29.7% (110 participants), cleaned their brushes monthly, with 10.8% (40 participants) opting for a weekly cleaning regimen. The majority, accounting for 44.3% (164 participants), rarely cleaned their makeup brushes. Concerning skin problems potentially linked to makeup products, 27.8% (103 participants) reported having experienced issues such as acne, irritation, or bacterial infections. Among those, 3.0% (11 participants) experienced facial infections more than twice per week, 7.6% (28 participants) experienced them several times per month, 12.4% (46 participants) experienced them monthly, and 31.4% (116 participants) experienced them sporadically every few months. A larger segment, 53.2% (197 participants), reported no such issues, while 18.9% (70 participants) were unsure if their skin problems were related to makeup use.

**Figure 6:**
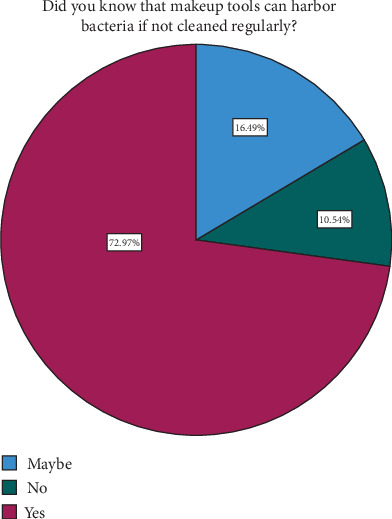
Awareness about makeup brush hygiene.

**Figure 7:**
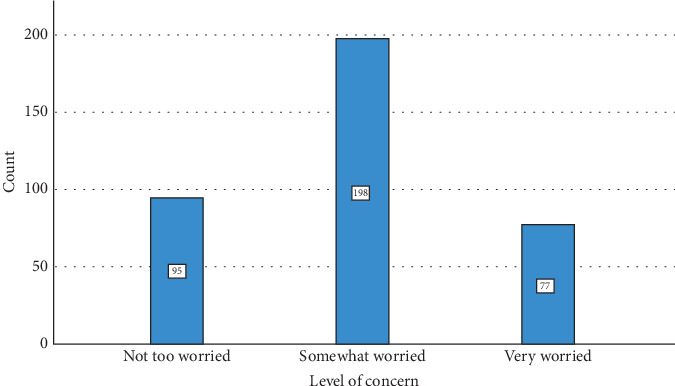
Level of concern about makeup brush hygiene.

**Figure 8:**
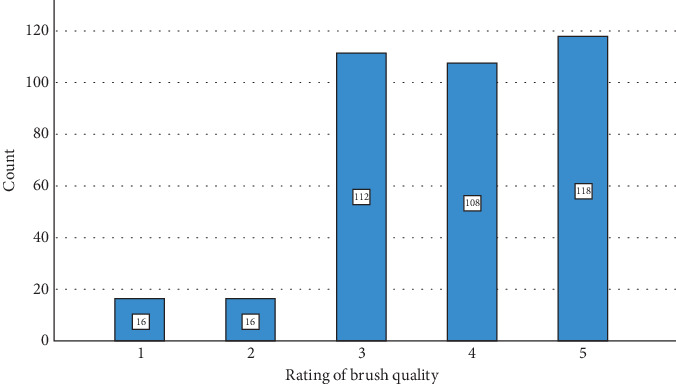
Quality of makeup brushes.

**Figure 9:**
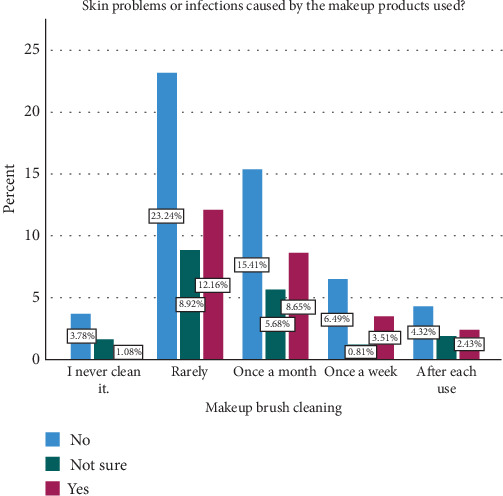
Association of makeup brush cleaning with skin problems and infections.

**Figure 10:**
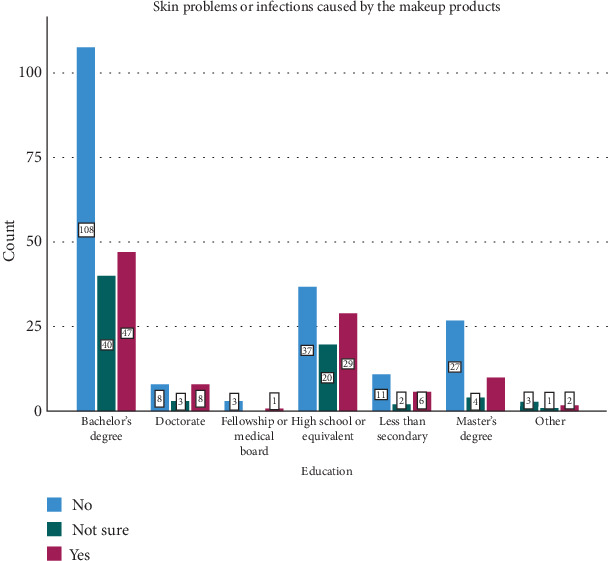
Difference in the skin problems caused by the level of education.

The survey findings from Table 5 and Figures 6, 7, and 8 depict different levels of knowledge, awareness, concerns, and ratings related to makeup tools among participants. A majority, 73.0% (270 participants), indicated that they were aware that makeup tools such as brushes and sponges can harbor bacteria if not cleaned regularly. Additionally, 16.5% (61 participants) were unsure or believed it was possible, while 10.5% (39 participants) were unaware of this fact. Concerning bacterial infections or skin problems from makeup tools, participants showed diverse levels of worry: 25.7% (95 participants) were not too worried, 53.5% (198 participants) were somewhat worried, and 20.8% (77 participants) were very worried. The ratings of beauty tools used in their care routines varied: 4.3% (16 participants) rated their tools as 1 or 2, 30.3% (112 participants) rated them as 3, and, nearly equally, 29.2% (108 participants) rated them as 4, while 31.9% (118 participants) gave a rating of 5, indicating high satisfaction.  Table 6 and Figure 9 examine the relationship between participants' cleaning habits with makeup brushes and the occurrence of skin problems or infections caused by makeup products. Among those who cleaned their brushes after each use, 8.1% (16 participants) reported no skin problems, 10.0% (seven participants) were unsure, and 8.7% (nine participants) experienced skin problems. For participants who admitted to never cleaning their brushes, 7.1% (14 participants) reported no skin issues, 8.6% (six participants) were unsure, and only 3.9% (four participants) experienced skin problems. Cleaning brushes once per month correlated with 28.9% (57 participants) reporting no skin problems, 30.0% (21 participants) being unsure, and 31.1% (32 participants) experiencing skin issues. Cleaning weekly showed 12.2% (24 participants) reporting no issues, 4.3% (three participants) being unsure, and 12.6% (13 participants) experiencing problems. Among those who rarely cleaned their brushes, 43.7% (86 participants) reported having no skin problems, 47.1% (33 participants) were unsure, and 43.7% (45 participants) reported having skin problems. The *p* value of 0.698 indicated that there was no statistically significant association between the frequency of makeup brush cleaning and the likelihood of experiencing skin problems or infections caused by makeup products among the participants.  Table 7 presents the results of the association between awareness of cleaning makeup brushes and the occurrence of skin problems among respondents. The *p* values associated with these findings indicate significant correlations between awareness levels and concerns about potential skin issues caused by makeup tools. Specifically, *p* values of 0.040 and 0.001 were noted in associations between awareness of cleaning practices and the concern for bacterial infections or skin problems, respectively.  Table 8 and Figure 10 present the results of a survey examining the relationship between education levels and makeup brush hygiene and awareness. A borderline significant association (*p* = 0.060) was found between education levels and cleaning frequency. Regarding the awareness of the potential bacterial accumulation in makeup tools, the responses were fairly consistent across educational backgrounds, with the *p* values suggesting no significant differences (0.941). The levels of concern about bacterial infections or skin problems caused by using makeup brushes and sponges also showed no significant differences across education levels (*p* = 0.404). Participants were also asked to rate the quality of their beauty tools on a scale from 1 to 5, where 1 represents the *lowest quality* and 5 represents the *highest*. The ratings varied across educational groups but did not show statistically significant differences (*p* = 0.115).

## 4. Discussion

Various studies have shown that makeup tools, including brushes and beauty blenders, can harbor a range of bacteria, including opportunistic pathogens. In the analysis of microbial isolates from cosmetics, a significant predominance of Gram-positive bacteria (81%) over Gram-negative bacteria (19%) was observed. This pattern is consistent with findings from other studies examining microbial contamination in cosmetics and personal care products, where Gram-positive bacteria often dominate due to their resilience and compatibility with the lipid-rich and preservative-containing formulations of these products [21, 22].

Since their thick peptidoglycan cell wall provides defense against environmental stressors such as preservatives and low-moisture conditions, Gram-positive bacteria, especially those belonging to genera such as *Staphylococcus*, *Micrococcus*, and *Bacillus*, are well suited to thrive in cosmetic environments [23]. The ability of certain Gram-positive species to form biofilms, which can shield them from antimicrobial compounds frequently found in personal care products, further increases their resilience [24].

Gram-negative bacteria are less common in cosmetics (19%) than Gram-positive bacteria, most likely because of their weaker cell wall structure and generally greater sensitivity to preservatives. Though they can survive in watery or preservative-deficient formulations, Gram-negative species such as *Pseudomonas* and *Ralstonia*, which are prevalent in cosmetics, are frequently noteworthy for their adaptability and resistance to certain antimicrobials [25, 26].

This distribution has implications for quality control and product formulation in the cosmetic industry. Since Gram-positive bacteria are more resilient to certain preservatives, there is a need to employ broad-spectrum antimicrobial agents or preservative systems that are effective against both Gram-positive and Gram-negative bacteria to ensure product safety [27].

The analysis of 16S rRNA gene sequences from cosmetic product samples reveals a notable diversity of bacterial families, suggesting a complex microbial presence that may arise from manufacturing processes, raw materials, or environmental contamination. This broad diversity underscores both the environmental resilience of certain bacterial families and potential quality control concerns for cosmetics [24, 27].

Micrococci (*n* = 22) were the most prevalent isolates. Species within this genus are commonly found on human skin and in various environmental settings. Although usually nonpathogenic, certain strains can cause opportunistic infections, particularly in individuals with compromised immunity [28]. The staphylococci (*n* = 16), another common group in skin microbiota, includes both harmless species and pathogens such as *Staphylococcus aureus*, a frequent cause of skin infections and a possible contaminant in personal care products [23]. Studies have revealed that *Staphylococcus aureus* can be found in considerable amounts in cosmetic blenders and makeup brushes. For example, a study found that the average bacterial load in cosmetic blenders was more than 10^6^ CFU/mL, and that one of the pathogens found was *Staphylococcus aureus* [29].

This implies that adding these strains to cosmetics may improve their ability to support skin health. Micrococci may have advantages, but they can also be a sign of contamination in cosmetics. Because cosmetics are frequently nutrient-rich settings, microbial growth may be encouraged, resulting in spoiling and possible health hazards in the event that pathogenic strains are present [30].

Bacilli (*n* = 11), known for their spore-forming ability, are highly resilient and capable of surviving in diverse conditions. This characteristic can make bacilli a recurring group in cosmetics, as they withstand environmental stressors and have even been found in preservative-free products [22].

*Bacillus subtilis* was found as one of the most predominant species in cosmetics representing 12% of all contaminant bacteria [30]. Some *Bacillus* species are beneficial, while others can be pathogenic, raising concerns about their presence in cosmetic products and tools [30].

*Ralstonia* (*n* = 7), a less common genus in such environments, includes species such as *Ralstonia pickettii*, which can thrive in water-based environments and is sometimes linked to contamination in pharmaceutical products due to its adaptability [26]. *Ralstonia pickettii* in cosmetic products can be harmful to one's health, especially for those with compromised immune systems. Skin irritations or infections can result from the contamination of skin care products by this bacterium, which has been linked to opportunistic infections [23, 31].

Isolates of *Exiguobacterium* (*n* = 3), *Sediminibacterium* (*n* = 2), *Pseudomonas* (*n* = 2), and *Pantoea* (*n* = 2) add to the diversity found in the samples. There is not much precise information on *Exiguobacterium acetylicum* in relation to makeup tools at the moment. *Exiguobacterium*, which includes *Exiguobacterium acetylicum*, has been researched for its possible uses in a number of domains, such as biotechnology and the creation of natural products, which may have an indirect connection to cosmetic formulations [32].

The *Sediminibacterium* genus of bacteria has been researched for its possible uses in cosmetics and other sectors. Although there are not many particular findings on *Sediminibacterium* in makeup brushes and beauty blenders, its traits and qualities point to potential advantages in cosmetic compositions. According to research, the skin microbiota is essential for preserving the health of the skin. Skin disorders and general skin health can be influenced by the presence of different microbial communities, which include bacteria such as *Sediminibacterium*. For example, a study on the microbiota of the skin discovered that some bacterial taxa, such as *Sediminibacterium*, can change depending on things like personal hygiene habits and cosmetic product use [33].

Pseudomonads are particularly known for their metabolic versatility and ability to thrive in various environments, including cosmetic products, which often contain organic compounds suitable for bacterial growth [25]. Pseudomonad contamination of makeup brushes and beauty blenders is a major concern. Numerous skin infections and other health problems can be caused by opportunistic microorganisms called pseudomonads, including *Pseudomonas aeruginosa* [34].

The available literature currently does not contain any particular reports describing the isolation of *Pantoea septica* from makeup tools. Without identifying *Pantoea septica*, the majority of research focuses on other *Pantoea* species or the overall microbiological quality of cosmetics. Because *Pantoea septica* has been linked to illnesses in immunocompromised people, it is crucial to make sure that makeup tools are free of dangerous microbes. Consumers who have sensitive skin or underlying medical disorders may be at risk, as indicated by the isolation of such bacteria from makeup tools [35].

Various studies have shown that makeup tools, including brushes and beauty blenders, can harbor a range of bacteria, including opportunistic pathogens. *Acinetobacter* (*n* = 1), *Glutamicibacter* (*n* = 1), *Robertmurraya* (*n* = 1), *Saprospiraceae* (*n* = 1), *Mixta* (*n* = 1), and *Arthrobacter* (*n* = 1) were each represented by one isolate. Many of these genera, such as *Acinetobacter*, are recognized for their role in biofilm formation and resistance to preservatives, which pose challenges for maintaining sterility in makeup tools [36].

Some *Acinetobacter* species are opportunistic pathogens, which raises safety concerns in cosmetic formulations. However, the presence of *Acinetobacter*, *Glutamicibacter*, *Robertmurraya*, *Saprospiraceae*, *Mixta*, and *Arthrobacter* in makeup tools is not well-documented, but contamination could occur. *Acinetobacter* can be separated from beauty blenders and cosmetic brushes, which puts people at risk for skin infections, particularly those with weakened immune systems [37].

The variety of bacterial species that can infect cosmetics, often from environmental sources or contamination during handling, is highlighted by these findings. In order to guarantee consumer safety and product lifetime, this microbial variety highlights the necessity for enhanced preservation techniques and quality control procedures [21].

The study participants' sociodemographic profile, which is shown in Table 1, offers important insights into the traits of cosmetic users, such as their age distribution, educational background, and makeup tool usage patterns. In the cosmetics sector, these kinds of data are crucial for comprehending customer behavior and can direct consumer education, focused marketing, and product development.

Individuals between the ages of 35 and 54 were significantly represented in the study, with 28.9% (107 individuals) being between the ages of 35 and 44 and 21.1% (78 participants) being between the ages of 45 and 54. According to Chin and Harun and Sinha and Jha, this age group frequently exhibits established consumer behaviors and financial stability, which are linked to higher expenditure on cosmetics and personal care [32, 33]. Younger age groups, such as those under 18 (3.0% or 11 participants) and those between 18 and 24 (11.6% or 43 participants), on the other hand, also made significant contributions, suggesting that younger consumers are interested in cosmetics, which is probably due to the impact of social media and beauty trends [34]. Participants over 65 made up 4.3% (16 participants), and those between the ages of 55 and 64 made up 16.8% (62 participants). This indicates that people continue to use cosmetics into later adulthood, frequently concentrating on skincare and antiaging products [35].

According to Ghazali et al., the majority of participants (52.7%) had bachelor's degrees, suggesting that the sample was reasonably educated and probably aware of product contents, ethical sourcing, and quality standards [36]. A sizable percentage of participants had only completed high school (23.2% or 86 participants), demonstrating the appeal of cosmetics to people of all educational backgrounds. Master's degrees (11.1% or 41 participants) and doctorates (5.1% or 19 participants) were held by a smaller but noteworthy proportion, indicating that higher educational attainment may be associated with particular preferences, such as “clean beauty” and cruelty-free products [21]. Because of their professional backgrounds, specialized credentials such as fellowships or medical board certifications (1.1% or 4 participants) may affect how people view the safety, effectiveness, and constituents of a product.

A range of participants' makeup tool usage frequencies are shown in Table 2. A significant percentage (31.1% or 115 participants) said that they hardly ever used makeup equipment, indicating a tendency toward infrequent or minimalist makeup practices [38]. On the other hand, daily users made up 20.3% of the sample (75 participants), indicating a group that is more committed to applying makeup on a regular basis. Moderate engagement with cosmetic regimens was demonstrated by participants who used tools once per week (16.5% or 61 participants) and several times per week (30.8% or 114 participants). Cosmetic consumers use makeup products extensively, as evidenced by the fact that only 1.4% (five participants) said that they did not use them [33].

Different beauty practices and product preferences are highlighted by the particular makeup brush types utilized by the participants. The prevalence of blush as a mainstay in cosmetic routines was shown by the fact that 88.6% of participants (328) reported using blush brushes. The preference for multipurpose tools was demonstrated by the following: eyeshadow brushes (60.0% or 222 participants) and powder brushes (46.8% or 173 participants). Foundation brushes (48.4% or 179 participants) were also widely used, particularly for even skin coverage, while contour brushes were used by 26.8% (99 participants), suggesting some engagement with advanced application techniques. Eyeliner brushes (25.1% or 93 participants) and lip brushes (16.5% or 61 participants) were less common, which was likely due to the popularity of alternative applicators. Only 4.1% (15 participants) reported not using any makeup brushes, reinforcing the importance of tools in modern cosmetic routines [23, 26].

These sociodemographic findings show that a wide range of age and educational categories, each with its own usage patterns and preferences, make extensive use of cosmetics and makeup products. Brands looking to target particular demographic groups can utilize this information to find market opportunities, such as products for younger customers interested in social media trends or elderly consumers interested in skincare and antiaging products. The kinds of tools and cosmetics that consumers like may also be influenced by education and product awareness, with educated consumers showing a preference for sustainable and ethically based goods [35].

The information reveals a broad variety of cleaning practices for sponges and cosmetic brushes. Just 8.6% of the participants (32 people) cleaned their equipment after every use, indicating a high level of hygiene knowledge that may help lower the risk of skin infections and bacterial accumulation [36]. Conversely, 24 participants, or 6.5% of the sample, acknowledged that they never cleaned their instruments, a habit that might lead to contamination and skin problems [23]. In addition, 10.8% (40 participants) chose weekly brush cleaning, whereas a larger group (29.7% or 110 participants) chose monthly brush cleaning. The majority (44.3% or 164 participants) reported rarely cleaning their makeup tools, indicating a common lack of consistent maintenance, which can increase the risk of microbial contamination and skin irritation [21, 38].

Given the known link between dirty makeup tools and the spread of bacteria, especially *Staphylococcus* and *Pseudomonas* species, which are known to colonize cosmetic products and tools, these findings imply that although a subset of users regularly cleans, the majority do not follow strict hygiene practices [26].

A significant proportion of participants (27.8% or 103 participants) reported experiencing skin problems potentially linked to makeup, including acne, irritation, and bacterial infections. Of these individuals, a small fraction (3.0% or 11 participants) faced recurring infections more than twice per week, while others experienced issues several times per month (7.6% or 28 participants) or monthly (12.4% or 46 participants). The largest subgroup within those experiencing skin issues, 31.4% (116 participants), reported sporadic problems every few months, suggesting that occasional exposure to unclean tools or contaminated products may exacerbate skin issues [39]. Conversely, 53.2% (197 participants) reported no skin problems, and 18.9% (70 participants) were uncertain if their skin concerns were related to cosmetics, which may indicate unrecognized or underlying sensitivities to product ingredients or contaminants [34].

These results demonstrate the potential for hygiene education related to the upkeep of makeup tools. To reduce bacterial contamination, which can cause infections and skin irritation, makeup brushes and sponges must be cleaned on a regular basis [26]. Given the high frequency of uncleaned equipment, skin problems associated with makeup are alarming and highlight the need for increased knowledge of product safety and upkeep. It may be possible to lower the prevalence of skin issues linked to cosmetics and promote safer beauty practices by educating consumers, especially those in the “rarely clean” category, about the value of routine cleansing.

According to Table 4, the vast majority of participants (270 or 73.0%) knew that makeup equipment could harbor bacteria if not cleaned on a regular basis. This supports other studies that demonstrated the public's understanding of the significance of hygiene in cosmetics, but it also shows that a sizeable percentage, 27.0%, were either ignorant or unsure [34]. While 53.5% of respondents were “somewhat worried”, just 20.8% were strongly concerned about possible infections or skin issues brought on by makeup equipment. Although individual understanding varies, these findings might be a reflection of consumers' increasing awareness of the importance of hygiene in preventing illnesses linked to cosmetics [23].

When asked to rank the perceived quality of their beauty equipment, 31.9% of participants gave them a high satisfaction rating of 5. However, previous research indicates that high tool satisfaction frequently does not translate into regular cleaning behaviors; therefore, this pleasure does not necessarily correlate with effective tool maintenance [36].

The association between participants' cleaning practices and the prevalence of skin issues is examined in Table 5. A tiny percentage of people who cleaned their brushes after every use experienced skin issues, which suggests that regular cleaning may reduce the risk of infections, as suggested by the literature [21]. Participants who cleaned their instruments infrequently or never at all, on the other hand, displayed greater doubt or a higher frequency of skin problems, indicating that irregular cleaning may increase exposure to germs and other pathogens. Nevertheless, a *p* value of 0.698 indicates that there is no statistically significant correlation between the frequency of cleaning and the development of skin issues, suggesting that infection rates may be influenced by variables other than cleaning frequency.

Table 6 shows that there are considerable connections between concerns about bacterial illnesses and awareness of hygiene procedures, with *p* values of 0.040 and 0.001. This result implies that participants are more likely to worry about infections if they are aware of the possible dangers of using dirty makeup products. These conclusions are supported by the literature, which demonstrates that heightened knowledge of the advantages of hygiene can have a good impact on personal care practices and can reduce the likelihood of skin problems [39].

An examination of education levels in connection to awareness and cleanliness habits is shown in Table 7. Concern about infections and awareness of bacterial accumulation did not differ significantly among education groups, despite a marginally significant correlation (*p* = 0.060) between cleaning frequency and education levels. Because hygiene information is widely disseminated through a variety of media, this suggests that understanding of cleanliness practices transcends educational backgrounds [38].

When used, cosmetic brushes and beauty blenders come into contact with the skin's surface, oils, germs, and makeup products. These tools are often used, which exposes them to a variety of diseases that, if not properly cleaned, can grow and spread. Particularly when used moist, beauty blenders provide the perfect conditions for the growth of bacteria. Remaining moisture after use creates an ideal environment for microorganisms to grow if it is not adequately dried. In a similar vein, if brushes are not sufficiently dried after washing, they may harbor bacteria. Cosmetic users' awareness of proper hygiene measures is one of the most important aspects in preventing bacterial contamination on makeup instruments. The majority of users (73%) are aware of the significance of keeping instruments clean and the possible repercussions for their skin health, despite the established concerns. Many users either do not clean their makeup equipment on a regular basis or, when they do, they might not employ the right cleaning methods. Only 32% of users cleaned their beauty blenders and brushes on a regular basis, according to a study by [40]. A sizable portion of participants rarely (44.3%) cleaned their brushes and blenders or only once a month (29.7%). Frequent cleaning raises the risk of bacterial growth, which can cause discomfort and skin problems like acne. Many makeup users do not associate the use of contaminated makeup products with skin conditions including redness, rashes, or acne. This ignorance of the dangers of dirty tools can result in recurrent exposure to dangerous microorganisms. According to Michalek et al. [41], a large number of customers were ignorant of the microbiological hazards connected to unclean makeup equipment, which exacerbated skin issues.

## 5. Conclusions and Implications

The study reveals a complex relationship between cosmetic users' awareness, hygiene habits, and skin issues. While no direct link was found between cleaning frequency and reported problems such as acne, irritation, or infections, bacterial contamination remains a concern. Other factors, including improper storage, brush-sharing, and infrequent disinfection, may play a greater role in microbial exposure and skin reactions. Interestingly, individuals with higher awareness of hygiene risks exhibit greater concern about potential infections, yet their cleaning frequency remains inconsistent. This highlights a gap between awareness and action. Therefore, educational efforts should focus on promoting behavioral changes rather than merely increasing awareness. Targeted campaigns should encourage consistent tool maintenance, particularly for infrequent cleaners, regardless of educational background. Since education level only marginally influences cleaning frequency, interventions should prioritize simple, practical hygiene practices. Further research should explore how hygiene behaviors, bacterial contamination, and skin health outcomes are interconnected.

## Figures and Tables

**Table 1:** The diverse bacterial strains isolated from makeup brushes and beauty blenders, with *Micrococcus*, *Staphylococcus*, and *Bacillus* being the most prevalent. These bacteria, commonly found on human skin, can contribute to opportunistic infections and contamination. The presence of *Ralstonia*, *Pseudomonas*, and *Acinetobacter* suggests environmental contamination, reinforcing the importance of regular cleaning to maintain hygiene and reduce skin-related risks.

**Family**	**Species**
*Bacillus* (*n* = 11)	*Bacillus cereus* *Bacillus* sp. (in: *Firmicutes)* *Bacillus amyloliquefaciens* *Bacillus atrophaeus* *Bacillus velezensis* *Bacillus subtilis* *Bacillus velezensis*

*Exiguobacterium* (*n* = 3)	*Exiguobacterium acetylicum* *Exiguobacterium* sp. *Exiguobacterium indicum*

*Ralstonia* (*n* = 7)	*Ralstonia* sp. *Ralstonia pickettii*

*Mixta* (*n* = 1)	*Mixta calida*

*Acinetobacter* (*n* = 1)	*Acinetobacter calcoaceticus*

*Glutamicibacter* (*n* = 1)	*Glutamicibacter soli*

*Arthrobacter* (*n* = 1)	*Arthrobacter* sp.

*Staphylococcus* (*n* = 16)	*Staphylococcus pasteuri* *Staphylococcus aureus* *Staphylococcus haemolyticus* *Staphylococcus epidermidis* *Staphylococcus* sp. *Staphylococcus warneri* *Staphylococcus hominis* *Staphylococcus saprophyticus*

*Micrococcus* (*n* = 22)	*Micrococcus luteus* *Micrococcus aloeverae* *Micrococcus* sp. *Micrococcus yunnanensis*

*Pseudomonas* (*n* = 2)	*Pseudomonas stutzeri* *Pseudomonas putida*

*Pantoea* (*n* = 2)	*Pantoea septica*

*Robertmurraya* (*n* = 1)	*Robertmurraya korlensis*

*Saprospiraceae* (*n* = 1)	*Saprospiraceae bacterium*

*Sediminibacterium* (*n* = 2)	*Sediminibacterium* sp.

**Table 2:** Sociodemographic characteristics of the participants.

		**Count**	**%**
Age	Less than 18 years	11	3.0%
	18–24 years	43	11.6%
	25–34 years	53	14.3%
	35–44 years	107	28.9%
	45–54 years	78	21.1%
	55–64 years	62	16.8%
	More than 65 years	16	4.3%

Education	Less than secondary	19	5.1%
	High school or equivalent	86	23.2%
	Bachelor's degree	195	52.7%
	Master's degree	41	11.1%
	Fellowship or medical board	4	1.1%
	Doctorate	19	5.1%
	Other	6	1.6%

**Table 3:** Types of makeup brushes and frequency of use.

		**Count**	**%**
How often do you use makeup tools (brush, sponge, etc.)?	I do not use it	5	1.4%
	Rarely	115	31.1%
	Once a week	61	16.5%
	Every day	75	20.3%
	Several times a week	114	30.8%

Makeup products used	Powder brush	173	46.8%
	Blush brush	328	88.6%
	Eyeshadow brush	222	60.0%
	Foundation brush	179	48.4%
	Contour brush	99	26.8%
	Eyeliner brush	93	25.1%
	Lip brush	61	16.5%
	I do not use it	15	4.1%

**Table 4:** Cleaning habits of participants regarding makeup brushes.

		**Count**	**%**
How often do you clean a makeup brush and sponge?	After each use	32	8.6%
	I never clean it	24	6.5%
	Once a month	110	29.7%
	Once a week	40	10.8%
	Rarely	164	44.3%

Have you ever encountered any skin problems or infections (such as acne, irritation, and bacterial infections) that you suspect may be caused by the makeup products used?	No	197	53.2%
	Not sure	70	18.9%
	Yes	103	27.8%

If the answer is yes, how often have you experienced facial infections or acne?	No	169	45.7%
	Continuously (more than twice a week)	11	3.0%
	Frequently (several times a month)	28	7.6%
	Sometimes (once a month)	46	12.4%
	Rarely (once every few months)	116	31.4%

**Table 5:** Knowledge and awareness of participants about the cleanliness of makeup brushes.

		**Count**	**%**
Did you know that makeup tools (such as brushes and sponges) can harbor bacteria if not cleaned regularly?	Maybe	61	16.5%
	No	39	10.5%
	Yes	270	73.0%

How concerned are you about bacterial infections or skin problems caused by using makeup brushes and sponges?	Not too worried	95	25.7%
	Somewhat worried	198	53.5%
	Very worried	77	20.8%

Please rate the quality of the beauty tools you use in your care routine.	1	16	4.3%
	2	16	4.3%
	3	112	30.3%
	4	108	29.2%
	5	118	31.9%

**Table 6:** Association of habits of cleaning makeup brushes and the causation of skin problems.

		**Skin problems or infections (such as acne, irritation, and bacterial infections) caused by the makeup products used**						**p** ** value**
		**No**		**Not sure**		**Yes**		
		**Count**	**%**	**Count**	**%**	**Count**	**%**	
How often do you clean a makeup brush and sponge?	After each use	16	8.1%	7	10.0%	9	8.7%	0.698
	I never clean it	14	7.1%	6	8.6%	4	3.9%	
	Once a month	57	28.9%	21	30.0%	32	31.1%	
	Once a week	24	12.2%	3	4.3%	13	12.6%	
	Rarely	86	43.7%	33	47.1%	45	43.7%	

**Table 7:** Association of the awareness of cleaning of makeup brushes and the causation of skin problems.

		**Skin problems or infections (such as acne, irritation, and bacterial infections) caused by the makeup products used**						**p** ** value**
		**No**		**Not sure**		**Yes**		
		**Count**	**%**	**Count**	**%**	**Count**	**%**	
Did you know that makeup tools (such as brushes and sponges) can harbor bacteria if not cleaned regularly?	Maybe	32	16.2%	18	25.7%	11	10.7%	0.040
	No	21	10.7%	3	4.3%	15	14.6%	
	Yes	144	73.1%	49	70.0%	77	74.8%	

How concerned are you about bacterial infections or skin problems caused by using makeup brushes and sponges?	Not too worried	65	33.0%	15	21.4%	15	14.6%	0.001
	Somewhat worried	104	52.8%	39	55.7%	55	53.4%	
	Very worried	28	14.2%	16	22.9%	33	32.0%	

Please rate the quality of the beauty tools you use in your care routine.	1	11	5.6%	2	2.9%	3	2.9%	0.064
	2	6	3.0%	5	7.1%	5	4.9%	
	3	58	29.4%	14	20.0%	40	38.8%	
	4	51	25.9%	28	40.0%	29	28.2%	
	5	71	36.0%	21	30.0%	26	25.2%	

**Table 8:** Difference in the level of awareness with the level of education.

		**Education**														**p** ** value**
		**Bachelor's degree**		**Doctorate**		**Fellowship or medical board**		**High school or equivalent**		**Less than secondary**		**Master's degree**		**Other**		
		**Count**	**%**	**Count**	**%**	**Count**	**%**	**Count**	**%**	**Count**	**%**	**Count**	**%**	**Count**	**%**	
How often do you clean a makeup brush and sponge?	After each use	12	6.2%	0	0.0%	1	25.0%	12	14.0%	2	10.5%	3	7.3%	2	33.3%	0.060
	I never clean it.	13	6.7%	4	21.1%	0	0.0%	4	4.7%	2	10.5%	1	2.4%	0	0.0%	
	Once a month	61	31.3%	4	21.1%	0	0.0%	25	29.1%	6	31.6%	13	31.7%	1	16.7%	
	Once a week	20	10.3%	6	31.6%	0	0.0%	7	8.1%	3	15.8%	4	9.8%	0	0.0%	
	Rarely	89	45.6%	5	26.3%	3	75.0%	38	44.2%	6	31.6%	20	48.8%	3	50.0%	

Did you know that makeup tools (such as brushes and sponges) can harbor bacteria if not cleaned regularly?	Maybe	35	17.9%	3	15.8%	0	0.0%	15	17.4%	4	21.1%	4	9.8%	0	0.0%	0.941
	No	20	10.3%	3	15.8%	0	0.0%	9	10.5%	2	10.5%	4	9.8%	1	16.7%	
	Yes	140	71.8%	13	68.4%	4	100.0%	62	72.1%	13	68.4%	33	80.5%	5	83.3%	

How concerned are you about bacterial infections or skin problems caused by using makeup brushes and sponges?	Not too worried	41	21.0%	5	26.3%	1	25.0%	22	25.6%	7	36.8%	16	39.0%	3	50.0%	0.404
	Somewhat worried	109	55.9%	10	52.6%	3	75.0%	48	55.8%	8	42.1%	19	46.3%	1	16.7%	
	Very worried	45	23.1%	4	21.1%	0	0.0%	16	18.6%	4	21.1%	6	14.6%	2	33.3%	

Please rate the quality of the beauty tools you use in your care routine.	1	6	3.1%	2	10.5%	0	0.0%	2	2.3%	4	21.1%	2	4.9%	0	0.0%	0.115
	2	4	2.1%	0	0.0%	0	0.0%	7	8.1%	2	10.5%	2	4.9%	1	16.7%	
	3	65	33.3%	4	21.1%	2	50.0%	24	27.9%	4	21.1%	11	26.8%	2	33.3%	
	4	58	29.7%	6	31.6%	1	25.0%	27	31.4%	5	26.3%	11	26.8%	0	0.0%	
	5	62	31.8%	7	36.8%	1	25.0%	26	30.2%	4	21.1%	15	36.6%	3	50.0%	

## Data Availability

The data that support the findings of this study are available on request from the corresponding author.

## References

[1] Onurdağ F. K., Özgen S., Abbasoğlu D. (2010). Microbiological Investigation of Used Cosmetic Samples. *Hacettepe University Journal of the Faculty of Pharmacy*.

[2] Dadashi L., Dehghanzadeh R. (2016). Investigating Incidence of Bacterial and Fungal Contamination in Shared Cosmetic Kits Available in the Women Beauty Salons. *Health Promotion Perspective*.

[3] Enemuor S., Ojih M., Isah S., Oguntibeju O. (2013). Evaluation of Bacterial and Fungal Contamination in Hairdressing and Beauty Salons. *African Journal of Microbiology Research*.

[4] Behravan J., Bazzaz F., Malaekeh P. (2005). Survey of Bacteriological Contamination of Cosmetic Creams in Iran (2000). *International Journal of Dermatology*.

[5] Anelich L., Korsten L. (1996). Survey of Micro*-*Organisms Associated With Spoilage of Cosmetic Creams Manufactured in South Africa. *International Journal of Cosmetic Science*.

[6] Draelos Z. D. (2001). Special Considerations in Eye Cosmetics. *Clinics in Dermatology*.

[7] Tharmila S., Jeyaseelan E. C., Thavaranjit A. (2012). Inhibitory Effect of Some Traditional Hair Washing Substances on Hair Borne Bacteria. *Der Pharmacia Lettre*.

[8] Hassan S. M., Hamad A. K., Shallal A. F., Abdullah S. M. (2018). Isolation of Pathogenic Microbes From Beauty Salons in Ranya, Iraq. *Gazi Medical Journal*.

[9] Naz S., Iqtedar M., Ul Ain Q., Aftab K. (2012). Incidence of Human Skin Pathogens From Cosmetic Tools Used in Beauty Saloons in Different Areas of Lahore, Pakistan. *Journal of Scientific Research*.

[10] Carøe T. K., Ebbehøj N. E., Agner T. (2017). Occupational Dermatitis in Hairdressers–Influence of Individual and Environmental Factors. *Contact Dermatitis*.

[11] Alharbi N., Alhashim H. M. (2021). Identification of Pathogenic Microbes in Tools of Beauty Salon in Jeddah City. *Biosciences Biotechnology Research Asia*.

[12] Stout J. E., Gadkowski L. B., Rath S., Alspaugh J. A., Miller M. B., Cox G. M. (2011). Pedicure-Associated Rapidly Growing Mycobacterial Infection: An Endemic Disease. *Clinical Infectious Diseases*.

[13] Edward S. M., Megantara I., Dwiyana R. F. (2015). Detection of Fungi in Hair-Brushes in Beauty Salons at Jatinangor. *Althea Medical Journal*.

[14] Ruddy M., Cummins M., Drabu Y. (2001). Hospital Hairdresser as a Potential Source of Crossinfection With mrSA. *Journal of Hospital Infection*.

[15] Janmohammadi F., Ghodous F., Daem R., Kayvan F. (2016). Evaluation of Bacterial and Fungal Contaminations in Barbershops in Kamyaran City, Iran-Summer 2015. *International Journal of Medical Research and Health Sciences*.

[16] Sekula S. A., Havel A., Otillar L. J. (2002). Nail Salons Can Be Risky Business. *Archives of Dermatological Research*.

[17] Chennoufi I. H., Zanane C., Ameslek M., Zahir H., El Louali M., Latrache H. (2023). Physicochemical Characterization of Reusable Facemasks and Theoretical Adhesion by a Challenged Bacterium. *Iranian Journal of Microbiology*.

[18] Kabara J. J., Orth D. S., Kabara J. J., Orth D. S. (1997). Preservative-Free and Self-Preserving Cosmetics and Drugs. *Principles for Product Preservation*.

[19] Al-Rawi A. M., Bahjat S. A., Al-Allaf M. A. A. (2018). Novel Natural Disinfectants for Contaminated Cosmetic Application Tools. *International Journal of Molecular Sciences*.

[20] Poddębniak P., Kalinowska-Lis U. (2024). A Survey of Preservatives Used in Cosmetic Products. *Applied Sciences*.

[21] Giorgio A., Miele L., De Bonis S. (2018). Microbiological Stability of Cosmetics by Using Challenge Test Procedure. *Journal of Pure and Applied Microbiology*.

[22] Otto M. (2020). Staphylococci in the Human Microbiome: The Role of Host and Interbacterial Interactions. *Current Opinion in Microbiology*.

[23] Jairoun A. A., Al-Hemyari S. S., Shahwan M., Zyoud S. H. (2020). An Investigation into Incidences of Microbial Contamination in Cosmeceuticals in the UAE: Imbalances between Preservation and Microbial Contamination. *Cosmetics*.

[24] Gomila M., Del Carmen G. M., Fernández-Baca V. (2013). Genetic Diversity of Clinical *Pseudomonas aeruginosa* Isolates in a Public Hospital in Spain. *BMC Microbiology*.

[25] Ryan M. P., Adley C. C. (2014). Ralstonia spp.: Emerging Global Opportunistic Pathogens. *European Journal of Clinical Microbiology & Infectious Diseases*.

[26] Abdel-Fatah M. A., Amin A., Elkady H. (2021). Industrial Wastewater Treatment by Membrane Process. *Membrane-Based Hybrid Processes for Wastewater Treatment*.

[27] Percival S. L., Emanuel C., Cutting K. F., Williams D. W. (2012). Microbiology of the Skin and the Role of Biofilms in Infection. *International Wound Journal*.

[28] Alharbi N. M., Alhashim H. M. (2021). Beauty Salons Are Key Potential Sources of Disease Spread. *Infection and Drug Resistance*.

[29] Alshehrei F. M. (2023). Isolation and Identification of Microorganisms Associated With High-Quality and Low-Quality Cosmetics From Different Brands in Mecca Region-Saudi Arabia. *Saudi Journal of Biological Sciences*.

[30] McLoughlin I. J., Voss A. L., Hale J. D., Jain R. (2024). Cosmetic Efficacy of the Topical Probiotic *Micrococcus luteus* Q24 in Healthy Human Adults. *Cosmetics*.

[31] Bashir A., Lambert P. (2020). Microbiological Study of Used Cosmetic Products: Highlighting Possible Impact on Consumer Health. *Journal of Applied Microbiology*.

[32] Lee J. E., Goh M. L., Mohd Noor M. N. B. (2019). Understanding Purchase Intention of University Students Towards Skin Care Products. *PSU Research Review*.

[33] Amberg N., Fogarassy C. (2019). Green Consumer Behavior in the Cosmetics Market. *Resources*.

[34] Pudaruth S., Juwaheer T. D., Seewoo Y. D. (2015). Gender-Based Differences in Understanding the Purchasing Patterns of Eco-Friendly Cosmetics and Beauty Care Products in Mauritius: A Study of Female Customers. *Social Responsibility Journal*.

[35] Saif G. B., Alsheikh O. A., Alkhudhayri N. (2023). Knowledge, Attitude, and Practice of Skin Care Among Elderly Patients in Riyadh, Saudi Arabia. *Cureus*.

[36] Ghazali E., Soon P. C., Mutum D. S., Nguyen B. (2017). Health and Cosmetics: Investigating Consumers’ Values for Buying Organic Personal Care Products. *Journal of Retailing and Consumer Services*.

[37] Gedefie A., Demsis W., Ashagrie M. (2021). *Acinetobacter baumannii* Biofilm Formation and Its Role in Disease Pathogenesis: A Review. *Infection and Drug Resistance*.

[38] Byrne A., Milestone K. (2023). ‘He Wouldn’t Be Seen Using It…’Men’s Use of Male Grooming Products As a Form of Invisible Consumption. *Journal of Consumer Culture*.

[39] Baltacı D. Ç., Durmaz Y., Baltacı F. (2025). The Mediating Role of Attitudes in the Effect of Human and Environment-Centered Value Orientation on Green Cosmetic Product purchasing Behavior: Comparison of Different Countries. *Environment, Development and Sustainability*.

[40] Abbasi R., Bano S., Tunio S. A., Brohi N. A., Siddiqui A. (2024). Evaluating the Bacterial Contamination in Used Cosmetic Products: A Potential Threat to Consumer’s Health. *Proceedings of the Pakistan Academy of Sciences: B. Life and Environmental Sciences*.

[41] Michalek I. M., John S. M., Caetano dos Santos F. L. (2019). Microbiological Contamination of Cosmetic Products–Observations From Europe, 2005–2018. *Journal of the European Academy of Dermatology and Venereology*.

